# Low-carbohydrate diet score and risk of breast cancer: findings from a prospective cohort study

**DOI:** 10.1007/s12282-026-01826-7

**Published:** 2026-02-04

**Authors:** Yen Thi-Hai Pham, Renwei Wang, Jian-Min Yuan, Hung N. Luu

**Affiliations:** 1 University of Pittsburgh Medical Center Hillman Cancer Center, UPMC Cancer Pavilion, Suite 4C, Room 470, 5150 Centre Avenue, Pittsburgh, PA 15232 USA; 2 Department of Epidemiology, School of Public Health, University of Pittsburgh, Pittsburgh, PA USA; 3 Dr. Mary and Ron Neal Cancer Center, Houston Methodist Research Institute, Room R9-213, 6670 Bertner Avenue, Houston, TX 77030 USA

**Keywords:** Low-carbohydrate diet score, Breast cancer risk, Prospective cohort study

## Abstract

**Background:**

A low carbohydrate diet (LCD) reflects a dietary pattern characterized by reduced carbohydrate intake and higher consumption of protein and fat. Evidence on the role of LCD and risk of breast cancer is inconclusive. We, therefore, prospectively examined the association between LCD scores and breast cancer risk in the Singapore Chinese Health Study (SCHS).

**Methods:**

We used data of 34,028 female participants in the SCHS, a prospective cohort study with subjects recruited in Singapore during 1993–1998 period. LCD scores were derived from the semi-quantitative food frequency questionnaire at baseline. Breast cancer cases were identified through record linkage with the Singapore cancer registry. Cox proportional hazard regression method was used to calculate hazard ratios (HRs) and 95% confidence intervals (CIs) for breast cancer in relation to LCD scores.

**Results:**

We identified 1,131 participants who developed breast cancer after 17.6 years of follow-up and 616,410 person-years. We found a modest inverse association between total LCD (HR_per-SD increment_ = 0.94, 95% CI: 0.89–1.00; *P*_*trend*_ = 0.05) and plant-based LCD scores with breast cancer risk (HR_per-SD increment_ = 0.94, 95% CI: 0.89–1.00; *P*_*trend*_ = 0.06). No association was found between animal-based LCD and risk of breast cancer (HR_per-SD increment_ = 0.96, 95% CI: 0.90–1.02, *P*_*trend*_ = 0.18).

**Conclusion:**

Our findings suggest that adherence to a LCD, particularly plant-based LCD may be protective against the development of breast cancer, highlighting the potential role of dietary modification in cancer prevention strategies.

**Supplementary Information:**

The online version contains supplementary material available at 10.1007/s12282-026-01826-7.

## Introduction

Breast cancer is the most diagnosed cancer (> 2.3 million new cases) and the leading cause of cancer-related death (< 660,000 deaths) among women worldwide. Its incidence has been steadily rising, particularly in low- and middle-income countries, due to changing reproductive patterns, lifestyle behaviors, and increased screening [1]. In the United States, the current incidence rate of breast cancer is 135.8 per 100,000 female with close to 320,000 new cases and more than 42,000 deaths each year [2]. While breast cancer is a heterogeneous disease with diverse molecular subtypes, several well-established risk factors contribute to its development. These include non-modifiable factors such as age, female sex, family history, and genetic mutations (e.g., *BRCA1/2*) [3, 4]. Reproductive factors also play a significant role in breast cancer, for instance early menarche, late menopause, nulliparity, late age at first childbirth, and lack of breastfeeding are all associated with increased risk due to prolonged estrogen exposure [5]. Modifiable risk factors also play a significant role in breast cancer risk, including obesity, physical inactivity, alcohol consumption, hormone replacement therapy, and certain dietary patterns [6–8]. Understanding the interplay between genetic, hormonal, and lifestyle factors is critical for developing effective strategies for breast cancer prevention, early detection, and personalized treatment.

A low-carbohydrate diet (LCD) is a dietary approach that restricts carbohydrate intake in favor of increased protein and/or fat consumption and has gained popularity for weight loss [9, 10]. While its short-term benefits for weight reduction are well established, its long-term impact on chronic diseases, including cancer, remains unclear [11]. The LCD score is a composite measure designed to capture adherence to a low-carbohydrate dietary pattern by evaluating the relative contributions of carbohydrate, fat, and protein intake [12]. Unlike analyses focused on single nutrients, the LCD score offers a holistic assessment of dietary balance. There are three types of LCD scores, including (1) the total LCD score, reflecting overall macronutrient intake from all sources; (2) the animal-based LCD score, based on nutrients derived from animal products; and (3) the plant-based LCD score, which considers nutrients primarily from plant-based sources.

Several studies have examined the association between low carbohydrate diets (or LCDs) and various health outcomes. In our previous analysis among the Singapore Chinese population, we found that higher LCD scores were positively associated with increased risks of hepatocellular carcinoma and colorectal cancer [13, 14]. and between animal-based LCD with risk of bladder cancer [15]. Similarly, data from the Japan Public Health Center-based Prospective Study (JPHC) showed that a higher animal-based LCD score was associated with an increased risks of overall cancer, colorectal cancer, and lung cancer, while a higher plant-based LCD score was associated with a reduced risk of gastric cancer [16]. In contrast, findings from the Prostate, Lung, Colorectal, and Ovarian (PLCO) Cancer Screening Trial suggested an inverse association between LCD and pancreatic cancer risk [17]. Besides cancer outcomes, LCDs have also been linked to metabolic health. Plant-based LCDs were associated with a lower risk of type 2 diabetes in women from the Nurses’ Health Study (NHS) and in Japanese men from the Japan Collaborative Cohort Study (JACC) [12, 18]. Conversely, data from the Melbourne Collaborative Cohort Study (MCCS) showed that higher total and animal-based LCD scores were associated with an increased risk of type 2 diabetes in the Australian population, an effect largely attributed to obesity, as well as among Iranian adults and U.S. men in the Health Professionals Follow-up Study (HPFS) [19–21]. Notably, a recent analysis from the NHS and HPFS cohorts also found that among individuals with type 2 diabetes, greater adherence to an LCD was associated with lower all-cause, cardiovascular, and cancer-related mortality [22].

Data on dietary LCD score and risk of breast cancer are sparse. To date, there is one epidemiologic study among participants in the Nurses’ Health Study in the US that reported a significantly lower risk of overall mortality among women with breast cancer who had greater adherence to overall (total) LCD and plant-based LCD scores but not animal-based CLD score [23]. We therefore aimed to investigate the association between LCD scores and risk of breast cancer incidence in a prospective cohort study in Asians, the Singapore Chinese Health Study (SCHS) consisting of more than 63,000 Chinese Singaporeans. We examined separately the effect of total LCD, animal-based- and plant-based LCD scores on risk of incident breast cancer, for the first time, in Asian women.

## Methods

### Study population

We used data from the Singapore Chinese Health Study (SCHS) for this analysis. The design and enrollment procedures of the SCHS have been described previously [24]. Briefly, the SCHS is an on-going population-based prospective cohort study of 63,257 Chinese men and women, aged 45–74 years old at the baseline. Participants were recruited between April 1993 and December 1998 and belonged to the two major Chinese dialect groups in Singapore, the Hokkiens and the Cantonese, originally from the Fujian and Guangdong provinces of southern China, respectively. They constituted the two largest dialect groups among Chinese immigrants in Singapore and originated from neighboring regions in southern China, allowing us to evaluate health patterns associated with these major cultural and geographical background within Singapore’s public-housing population [25]. This selection also captured the predominant Chinese ancestral origins relevant to the main objective of our study of understanding diet, lifestyle and disease risk. The SCHS was conducted in accordance with both the Declarations of Helsinki and Istanbul with all study participants providing informed consents. The SCHS study has been continuously approved by the Institutional Review Boards (IRBs) of the National University of Singapore and the University of Pittsburgh.

At baseline, trained interviewers conducted in-person home interview using structured questionnaires to collect information on demographics, body weight and height, family history of cancer, lifetime use of tobacco, menstrual/reproductive history (for women only), occupational exposure, current physical activity, and medical history. In the follow-up interview, the interviewers called the study participants to obtain updated information on alcohol use, tobacco smoking, medical history, current physical activity, and body weight and to invite participants to contribute blood and urine biospecimens for research purposes. Between July 1999 and December 2003, 28,346 participants (or about 57%) provided blood samples for research purposes.

### Dietary assessment

Dietary information in the SCHS was assessed using a semi-quantitative food frequency questionnaire (FFQ), which was developed and validated specifically for this population [26]. The FFQ included 165 commonly consumed food items or food groups among Chinese Singaporeans. Participants were asked to report their frequency of consumption using eight response categories ranging from “*never or hardly ever*” to “*two or more times a day*”, followed by portion size selection based on food photographs, depicting small, medium, and large servings. Nutrient intake was calculated using the Singapore Food Composition Database, enabling the calculation of average daily intake for approximately 100 nutrients and non-nutrient compounds [26].

A validation study of the FFQ was conducted between April 1994 and March 1997 among a random sample of 810 participants of the entire SCHS participants. Dietary data from the FFQ were compared to two 24-h dietary recalls (24-HDRs), with one conducted on a weekday and the other on a weekend, spaced roughly two months apart. The correlation coefficients for calorie-adjusted nutrient intakes between the FFQ and 24-HDRs ranged from 0.24 (for saturated fat intake among Hokkien women) to 0.73 (for saturated fat intake among Cantonese men) [26].

### Low-carbohydrate diet score (LCD) calculation

In this analysis, we calculated three LCD scores separately—total, animal-based, and plant-based LCD scores, following the methods that developed and validated in prior studies [12, 14, 27, 28]. Briefly, participants were ranked based on their daily caloric intake from carbohydrates, fats and proteins. These rankings were then divided into 11 equal groups for each macronutrient. For carbohydrate intake, individuals in the lowest intake group were assigned a score of 10 whereas those in the highest group received a score of 0. In contrast, for fat and protein intake, the lowest intake group were assigned a score of 0 and the highest intake group received a score of 10. The total LCD score was then calculated by summing the individual scores for carbohydrate, fat, and protein, resulting in a composite score ranging from 0 to 30 where higher scores indicate lower carbohydrate and higher fat and protein intakes. Similarly, the animal-based and plant-based LCD scores were constructed by evaluating fat and protein intakes specifically from animal and plant sources, respectively. These two scores reflect dietary patterns favoring either animal-or plant-derived macronutrients while maintaining a low carbohydrate profile.

### Assessment of other covariates

Body mass index (BMI) was calculated by dividing weight in kilograms by the square of height in meters. Smoking status was categorized as “never-smokers”, “current-smokers”, and “former smokers” [29]. For current and former smokers, additional details were collected, including the number of cigarettes smoked per day and the duration of smoking in years. Alcohol drinking status was classified into four groups: non-drinker, monthly drinker, weekly drinker, and daily drinker. Physical activity was assessed using eight-level continuous scale (ranging from never, 0.5–1, 2–3, 4–6, 7–10, 11–20, 21–30, and 31 h or more per week) for three types of activities: 1) strenuous sports (i.e., jogging, bicycling on hills, tennis, squash, swimming laps, or aerobics); 2) vigorous work (i.e., moving heavy furniture, loading or unloading trucks, shoveling, or equivalent manual labor), and 3) moderate activities (i.e., brisk walking, bowling, bicycling on level ground, tai chi, and chi kung) [30]. Coffee drinking status was categorized as non-drinker/monthly/weekly drinker, 1 cup/day, 2–3 cups/day and ≥ 4 cups/day. History of diabetes was obtained by asking participants whether they were told by a physician, and, if so, the age at first diagnosis. Age when menstrual period became regular was categorized into never, < 13, 13–14, 15–16, and ≥ 17 years of age. Number of children was grouped into non, 1–2, 3–4 and ≥ 5 children. Menopausal status was categorized into premenopausal and postmenopausal. Family history of breast cancer was grouped into “yes” versus “no”. The use of hormone replacement therapy was categorized into “yes” versus no.

### Ascertainment of breast cancer cases

Cancer diagnosis and deaths among study participants were identified through linkage with the Singapore Registry of Births and Deaths and the Singapore Cancer Registry, respectively. The Singapore Cancer Registry has been collecting comprehensive data on cancer diagnoses since 1968 [31]. As of December 31, 2015, less than 1% of participants (or 56 individuals) were lost to follow-up due to migration out of Singapore. For this analysis, breast cancer cases were identified using the International Classification of Diseases-Oncology, 2nd Edition (ICD-O-2) code C50.x. After excluding 1,936 participants with a history of cancer at baseline, the final analysis included 1,131 incident cases of breast cancer and 32,897 female participants without a cancer diagnosis at recruitment.

### Statistical analysis

We calculated frequencies for categorical variables and means and standard deviation (SD) for continuous variables. Differences between breast cancer cases and non-cases, as well as across categories of the LCD scores, were assessed using the chi-square ( χ^2^) test for categorical variables and the *t*-test for continuous variables. We defined quartiles of LCD scores based upon their distributions among the entire cohort. Person-years at risk for each participant were calculated from the date of the baseline interview (or enrollment) to the date of diagnosis of breast cancer, death, migration out of Singapore, or December 31, 2015, whichever occurred first.

For the main analysis, we performed Cox proportional hazards regression models to examine the association between the LCD scores and risk of breast cancer. Hazard ratios (HRs) and 95% confidence intervals (CIs) were calculated for breast cancer incidence across both quartiles and continuous scales of LCD scores. Multivariable models were adjusted for the following covariates, including age at recruitment (years), sex, dialect groups (Hokkien or Cantonese), education level (no formal education, primary, secondary or higher), year of enrollment (1993–1995 vs. 1996–1998), body mass index (BMI, kg/m^2^), alcohol intake (non-/monthly, weekly, or daily drinkers), age when period became regular, number of children, menopausal status, family history of breast cancer [32], and total energy intake. Overweight and obesity were defined as BMI ≥ 23 kg/m^2^ in accordance with World Health Organization (WHO) guidelines for Asian populations [33, 34].

Stratified analyses were further performed by menopausal status (i.e., premenopausal vs. postmenopausal), BMI (i.e., < 23 kg/m^2^ vs. ≥ 23 mg/m^2^), hormone replacement therapy (HRT) users (i.e., no vs yes) and family history of breast cancer (i.e., no vs. yes). In the sensitivity analysis, we removed breast cancer cases and person-years observed within the first two years of observation after the enrollment. We used the Schoenfeld residual test to evaluate the proportionality assumption for hazards over follow-up time and found no violation. We also tested the linear trend for breast cancer risk with LCD scores based on the ordinal values of their quartiles. The interactions in the LCD score-breast cancer risk association between different groups of selected factors (i.e., menopausal status, BMI, HRT usage and family history of breast cancer) were tested by including product terms of the LCD scores and the selected risk factors in the multivariable Cox regression models.

All statistical analyses were performed using the SAS version 9.4 computer software (SAS Institute Inc., Cary, NC). All *P* values presented are two-sided and *P-*values less than 0.05 were statistically significant, except in the cases of applying the Bonferroni correction tests.

### Data availability statement

The data that support the findings of this study are available on request from the corresponding author. The data are not publicly available due to privacy or ethical restrictions.

### Code availability statement

The underlying code for this study is not publicly available for proprietary reasons.

## Results

After a mean (standard deviation or SD) follow-up of 17.6 (5.3) years and a total follow-up time of 616,410 person-years, 1,131 incident cases of breast cancer were identified among 34,028 female participants who were free of cancer at baseline. The mean age (± SD) of cancer diagnosis was 56.2 (± 8.0).

Compared to female participants who remained free of breast cancer during the study period (or non-cases hereafter), breast cancer participants (or cases) had higher education level, were less likely to be Hokkien dialect or ever smokers, had more children and older age of having first child, and were less likely to have natural menopause (all *P*-values < 0.05). The distributions of weekly physical activity, alcohol consumption status, history of diabetes, coffee drinking status, family history of breast cancer, use of HRT, age at menarche, age when period became regular, age at menopausal, BMI level, total energy intake, daily intakes of fiber, total fat, animal fat, saturated fat, monounsaturated fat, polyunsaturated fat, and total protein were comparable between cases and non-cases (Table 1).

**Table 1:** Distribution of baseline characteristics among study participants, the Singapore Chinese Health Study, 1993–2015

Characteristics	Total # women in SCHS (N = 34,028)	Breast cancer (n = 1,131)	Non-cases (n = 32,897)	*P-value*
Age at recruitment, (Mean ± SD)	56.2 ± 8.0	56.1 ± 7.7	56.3 ± 8.0	< 0.0001
Highest level of education				
No formal education	13,700 (40.3)	366 (32.4)	13,334 (40.5)	< 0.0001
Primary school	13,283 (39.0)	455 (40.2)	12,828 (39.0)	
Secondary school or higher	7045 (20.7)	310 (27.4)	6735 (20.5)	
Dialect				
Cantonese	16,290 (47.9)	575 (50.8)	15,715 (47.8)	0.04
Hokkien	17,738 (52.1)	556 (49.2)	17,182 (52.2)	
Weekly physical activity^a^				
No	25,599 (75.2)	840 (74.3)	24,759 (75.3)	0.45
Yes	8429 (24.8)	291 (25.7)	8138 (24.7)	
Smoking status				
Never Smoker	31,062 (91.3)	1053 (93.1)	30,009 (91.2)	0.03
Ever Smoker	2966 (8.7)	78 (7.0)	2888 (8.8)	
Alcohol consumption				
Non-Drinker/Monthly drinker	32,515 (95.6)	1084 (95.8)	31,431 (95.5)	0.89
Weekly drinker	1121 (3.3)	35 (3.1)	1086 (3.3)	
Daily drinker	392 (1.1)	12 (1.1)	380 (1.2)	
History of diabetes				
No	30,919 (90.9)	1045 (92.4)	29,874 (90.8)	0.07
Yes	3109 (9.1)	86 (7.6)	3023 (9.2)	
Coffee drinking status				
Non-drinker/monthly/weekly	10,356 (30.4)	343 (30.3)	10,013 (30.4)	0.19
1 cup/day	13,644 (40.1)	431 (38.1)	13,213 (40.2)	
2–3 cups/day	9133 (26.8)	332 (29.3)	8801 (26.7)	
≥ 4 cups/day	895 (2.6)	25 (2.2)	870 (2.6)	
Family history of breast cancer				
No	33,590 (98.7)	1115 (98.6)	32,475 (98.7)	0.70
Yes	438 (1.3)	16 (1.4)	422 (1.3)	
Use of hormone replacement therapy				
No	32,106 (94.4)	1050 (92.8)	31,056 (94.4)	0.82
Yes	1922 (5.6)	81 (7.2)	1841 (5.6)	
Age at menarche (Mean ± SD)	14.4 ± 1.8	14.2 ± 1.7	14.4 ± 1.8	0.18
Age when period became regular (Mean ± SD)	14.1 ± 3.2	14.0 ± 2.9	14.1 ± 3.2	0.36
Number of children				
0	2388 (7.0)	114 (10.1)	2274 (6.9)	< 0.0001
1–2	9587 (28.2)	386 (34.1)	9201 (30.0)	
3–4	12,624 (37.1)	417 (36.9)	12,207 (37.1)	
≥ 5	9429 (27.7)	214 (18.9)	9215 (28.0)	
Age at first child (Mean ± SD)	24.6 ± 4.7	25.4 ± 4.9	24.6 ± 4.7	< 0.0001
Menopausal status				
Still menstruating	9593 (28.3)	379 (33.5)	9214 (28.0)	< 0.0001
Natural	21,341 (62.7)	637 (56.3)	20,704 (63.0)	
Other	3088 (9.1)	115 (10.2)	2973 (9.0)	
Age at menopausal (Mean ± SD)	49.6 ± 4.3	49.6 ± 4.3	49.3 ± 4.3	0.13
BMI, kg/m (Mean ± SD)	23.2 ± 3.3	23.5 ± 3.2	23.2 ± 3.3	0.08
Total Energy Intake (kcal/day)	1399.0 ± 472.6	1420.4 ± 467.5	1398.2 ± 472.8	0.12
Carbohydrate 1(gr/day) (Mean ± SD)	204.2 ± 67.0	208.7 ± 68.0	204.0 ± 67.0	0.02
Dietary fiber (gr/day) (Mean ± SD)	12.2 ± 5.6	12.4 ± 5.6	12.2 ± 5.6	0.15
Total fat (gr/day) (Mean ± SD)	40.6 ± 18.8	40.9 ± 18.4	40.6 ± 18.8	0.63
Animal fat (gr/day) (Mean ± SD)	13.1 ± 8.2	13.2 ± 8.1	13.1 ± 8.2	0.85
Plant fat (gr/day) (Mean ± SD)	27.5 ± 12.6	27.7 ± 12.1	27.5 ± 12.6	0.56
Saturated fat (gr/day) (Mean ± SD)	14.3 ± 7.2	14.2 ± 7.2	14.3 ± 7.2	0.95
Monounsaturated fat (gr/day) (Mean ± SD)	13.7 ± 6.5	13.7 ± 6.4	13.7 ± 6.5	0.77
Polyunsataturated fat (gr/day) (Mean ± SD)	8.3 ± 4.5	8.5 ± 4.4	8.3 ± 4.5	0.16
Total Protein (g/day) (Mean ± SD)	54.4 ± 21.5	54.8 ± 20.9	54.3 ± 21.5	0.46
Animal protein	29.4 ± 14.8	29.4 ± 14.4	29.3 ± 14.8	0.85
Plant protein	10.8 ± 6.5	10.8 ± 6.4	10.8 ± 6.4	0.95

^a^Including strenuous physical activity and/or vigorous work

Compared to those in the lowest quartile of total LCD score, participants with the highest quartile of total LCD score were younger, more likely to be female, having higher level of education and history of type 2 diabetes, but less likely to smoke cigarettes or drink alcohols. Additionally, they had higher intakes of calories, protein and fat but lower intakes of carbohydrate and were less likely to use HRT and less children. Similar patterns were observed for animal-based and plant-based LCD scores (Supplementary Table 1).

There was a modest inverse association between total LCD score (HR_per-SD increment_ = 0.93, 95% CI: 0.89–1.00; *P*_*trend*_ = 0.05) and a marginally significant association between plant-based LCD score and breast cancer risk {HR = 0.94, 95% CI: 0.89–1.00; *P*_*trend*_ = 0.06). Compared with the lowest quartile, the HRs (95% CIs) of breast cancer for quartiles 2, 3, and 4 of the total LCD score were 0.93 (0.79–1.10), 1.03 (0.87–1.22), and 0.81 (0.68–0.96), respectively; and of the plant-based LCD score were 1.04 (0.89–1.22), 0.92 (0.77–1.09) and 0.88 (0.74–1.04), respectively. No association between animal-based LCD score and risk of breast cancer was observed (HR _per-SD increment_ = 0.96, 95% CI: 0.90–1.02; *P*_*trend*_ = 0.18) (Table 2).

**Table 2:** Association between low-carbohydrate diet (LCD) score and risk of breast cancer: the Singapore Chinese Health Study, 1993–2015

Characteristics	Person-year	# breast cancer cases	HR^a^ (95% CI)
Total LCD score			
Q1 (lowest)	150,208	284	1.00
Q2	166,299	301	0.93 (0.79–1.10)
Q3	134,592	275	1.03 (0.87–1.22)
Q4 (highest)	165,311	271	0.81 (0.68–0.96)
*P*_*trend*_			0.05
Continuous scale (per SD increment)			0.94 (0.89–1.00)
Animal-based LCD score			
Q1 (lowest)	159,663	303	1.00
Q2	155,003	274	0.92 (0.78–1.08)
Q3	138,795	273	1.01 (0.85–1.19)
Q4 (highest)	162,950	281	0.86 (0.73–1.02)
*P*_*trend*_			0.18
Continuous scale (per SD increment)			0.96 (0.90–1.02)
Plant-based LCD score			
Q1 (lowest)	158,860	290	1.00
Q2	155,771	307	1.04 (0.89–1.22)
Q3	141,118	252	0.92 (0.77–1.09)
Q4 (highest)	160,662	282	0.88 (0.74–1.04)
*P*_*trend*_			0.06
Continuous scale (per SD increment)			0.94 (0.89–1.00)

*CI* confidence interval, *HR* hazard ratio, *LCD* low-carbohydrate diet

^a^Models adjusted for age at recruitment, education level, BMI (< 18.5, 18.5- < 23.0, 23.0- < 27.0, ≥ 27.0), dialect, year of enrollment, alcohol consumption status, number of children, menopausal status, age (year) when menstrual period became regular (< 13, 13–14, 15–16, or ≥ 17), family history of breast cancer, total energy intake

In stratified analysis, we did not find any difference in the associations for total LCD, animal-based and plant-based LCD scores with risk of breast cancer by menopausal status (yes, vs no), BMI level (< 23 vs ≥ 23 kg/m^2^, HRT use (yes vs no), or family history of breast cancer (yes vs no), except for plant-based LCD score in stratified analysis by family history of breast cancer (*P*_*heterogeneity*_ = 0.03) (Table 3).

**Table 3:** Association between low-carbohydrate diet score and risk of breast cancer, stratified by menopausal status, BMI levels, HRT usage status and family history of cancer: the Singapore Chinese Health Study, 1993-2015

		Q1	Q2	Q3	Q4	P_*Trend*_	Continuous scale (per SD increment)	*P*_*Interaction*_
Total LCD Score								
Pre-menopausal	# Cases	79	94	106	100			
	HR (95% CI)	1.00	0.83 (0.62–1.12)	1.03 (0.77–1.38)	0.68 (0.50–0.92)	0.04	0.90 (0.81–0.99)	0.21
Post-menopausal	# Cases	205	207	169	171			
	HR (95% CI)	1.00	0.98 (0.8–1.19)	1.02 (0.83–1.25)	0.89 (0.72–1.09)	0.36	0.97 (0.90–1.04)	
BMI < 23	# Cases	119	123	116	112			
	HR (95% CI)	1.00	0.86 (0.67–1.11)	0.99 (0.76–1.28)	0.75 (0.57–0.98)	0.08	0.92 (0.84–1.01)	0.76
BMI ≥ 23	# Cases	165	178	159	159			
	HR (95% CI)	1.00	0.99 (0.8–1.23)	1.07 (0.86–1.33)	0.87 (0.69–1.09)	0.31	0.96 (0.88–1.04)	
HRT non-users	# Cases	275	272	253	250			
	HR (95% CI)	1.00	0.88 (0.74–1.04)	1.00 (0.84–1.19)	0.80 (0.67–0.95)	0.05	0.94 (0.88–1.00)	0.98
HRT users	# Cases	9	29	22	21			
	HR (95% CI)	1.00	2.43 (1.15–5.15)	1.99 (0.91–4.34)	1.26 (0.57–2.79)	0.73	0.96 (0.77–1.20)	
Without family history of breast cancer	# Cases	277	298	272	268			
	HR (95% CI)	1.00	0.95 (0.81–1.12)	1.05 (0.89–1.25)	0.82 (0.69–0.98)	0.08	0.95 (0.89–1.01)	0.08
With family history of breast cancer	# Cases	7	3	3	3			
	HR (95% CI)	1.00	0.31 (0.08–1.23)	0.36 (0.09–1.44)	0.32 (0.08–1.35)	0.12	0.65 (0.38–1.11)	
Animal-based LCD score								
Pre-menopausal	# Cases	85	88	96	110			
	HR (95% CI)	1.00	0.85 (0.63–1.14)	0.94 (0.7–1.27)	0.81 (0.6–1.08)	0.25	0.94 (0.85- 1.04)	0.55
Post-menopausal	# Cases	218	186	177	171			
	HR (95% CI)	1.00	0.95 (0.78–1.15)	1.03 (0.85–1.26)	0.89 (0.72–1.09)	0.43	0.97 (0.90–1.04)	
BMI < 23	# Cases	130	111	113	116			
	HR (95% CI)	1.00	0.82 (0.64–1.06)	0.91 (0.70–1.17)	0.75 (0.58–0.98)	0.07	0.92 (0.83–1.01)	0.38
BMI ≥ 23	# Cases	173	163	160	165			
	HR (95% CI)	1.00	1.00 (0.8–1.23)	1.09 (0.87–1.35)	0.95 (0.76–1.19)	0.85	0.99 (0.92–1.07)	
HRT non-users	# Cases	288	253	249	260			
	HR (95% CI)	1.00	0.90 (0.76–1.07)	0.98 (0.83–1.17)	0.86 (0.73–1.03)	0.20	0.96 (0.90–1.02)	0.84
HRT users	# Cases	15	21	24	21			
	HR (95% CI)	1.00	1.15 (0.59–2.25)	1.40 (0.72–2.69)	0.87 (0.44–1.73)	0.72	0.96 (0.77–1.20)	
Without family history of breast cancer	# Cases	296	271	270	278			
	HR (95% CI)	1.00	0.93 (0.79–1.10)	1.02 (0.87–1.21)	0.88 (0.74–1.04)	0.25	0.96 (0.91–1.03)	0.09
With family history of breast cancer	# Cases	7	3	3	3			
	HR (95% CI)	1.00	0.24 (0.06–1.01)	0.31 (0.07–1.28)	0.28 (0.07–1.13)	0.08	0.62 (0.36–1.07)	
Plant-based LCD score								
Pre-menopausal	# Cases	86	94	89	110			
	HR (95% CI)	1.00	0.83 (0.62–1.12)	0.79 (0.59–1.07)	0.72 (0.54–0.96)	0.03	0.89 (0.80- 0.99)	0.17
Post-menopausal	# Cases	204	213	163	172			
	HR (95% CI)	1.00	1.14 (0.94–1.38)	0.97 (0.79–1.20)	0.96 (0.78–1.19)	0.46	0.97 (0.90–1.05)	
BMI < 23	# Cases	119	122	104	125			
	HR (95% CI)	1.00	0.96 (0.74–1.24)	0.88 (0.68–1.15)	0.87 (0.67–1.13)	0.25	0.94 (0.86–1.04)	0.70
BMI ≥ 23	# Cases	171	185	148	157			
	HR (95% CI)	1.00	1.10 (0.90–1.36)	0.95 (0.76–1.19)	0.89 (0.71–1.11)	0.17	0.94 (0.87–1.02)	
HRT non-users	# Cases	272	285	233	260			
	HR (95% CI)	1.00	1.04 (0.88–1.23)	0.93 (0.78–1.11)	0.89 (0.75–1.06)	0.11	0.95 (0.89–1.01)	0.32
HRT users	# Cases	18	22	19	22			
	HR (95% CI)	1.00	0.98 (0.52–1.82)	0.75 (0.39–1.44)	0.69 (0.36–1.31)	0.18	0.86 (0.69–1.07)	
Without family history of breast cancer	# Cases	283	304	249	279			
	HR (95% CI)	1.00	1.06 (0.9–1.25)	0.94 (0.79–1.11)	0.90 (0.75–1.06)	0.10	0.95 (0.89–1.01)	0.03
With family history of breast cancer	# Cases	7	3	3	3			
	HR (95% CI)	1.00	0.28 (0.07–1.10)	0.25 (0.06–1.01)	0.25 (0.06–1.09)	0.05	0.58 (0.33–1.00)	

Models adjusted for age at recruitment, education level, BMI (< 18.5, 18.5- < 23.0, 23.0- < 27.0, ≥ 27.0), dialect, year of enrollment, alcohol consumption status, number of children, menopausal status, age (year) when menstrual period became regular (< 13, 13–14, 15–16, or ≥ 17), family history of breast cancer, total energy intake

*CI* confidence interval, *HR* hazard ratio, *HRT* hormone replacement therapy, *LCD* low-carbohydrate diet

In the sensitivity analysis on the entire cohort after excluding breast cancer cases and person-years within the first two years of enrollment, the associations between total LCD and plant-based LCD scores with risk of breast cancer were not materially changed. Accordingly, the respective HRs and 95% CIs per SD of total LCD, animal-based LCD and plant-based LCD scores were 0.93 (0.88–1.00), 0.96 (0.90–1.02), and 0.93 (0.88–0.99). Also, compared to the lowest quartile (or quartile 1), the HRs and 95% CIs of breast cancer for the highest quartiles (or quartile 4) of total LCD, animal-based LCD and plant-based LCD scores were 0.79 (0.66–0.95), *P*_*trend*_ = 0.04, 0.85 (0.71–1.01), *P*_*trend*_ = 0.18, and 0.86 (0.71–1.02), *P*_trend_ = 0.03), respectively (Table 4**)**.

**Table 4:** Association between low-carbohydrate diet (LCD) score and risk of breast cancer after excluding incident cases and person-years in the first two years post-enrollment, the Singapore Chinese Health Study, 1993–2015

Characteristics	Person-year	# breast cancer cases	HR^a^ (95% CI)
Total LCD score			
Q1 (lowest)	133,507	262	1.00
Q2	148,071	276	0.93 (0.78–1.10**)**
Q3	119,911	256	1.04 (0.87–1.24)
Q4 (highest)	147,305	245	0.79 (0.66–0.95)
*P*_*trend*_			0.04
Continuous scale (per SD increment)			0.93 (0.88–1.00)
Animal-based LCD score			
Q1 (lowest)	142,037	280	1.00
Q2	137,986	248	0.90 (0.75–1.06)
Q3	123,671	255	1.01 (0.85–1.20)
Q4 (highest)	145,101	256	0.85 (0.71–1.01)
*P*_*trend*_			0.18
Continuous scale (per SD increment)			0.96 (0.90–1.02)
Plant-based LCD score			
Q1 (lowest)	141,063	269	1..0
Q2	138,716	282	1.03 (0.87–1.21)
Q3	125,775	233	0.91 (0.76–1.09)
Q4 (highest)	143,239	255	0.85 (0.71–1.02)
*P*_*trend*_			0.03
Continuous scale (per SD increment)			0.93 (0.88–0.99)

*CI* confidence interval, *HR* hazard ratio, *LCD* low-carbohydrate diet

^a^Models adjusted for age at recruitment, education level, BMI (< 18.5, 18.5- < 23.0, 23.0- < 27.0, ≥ 27.0), dialect, year of enrollment, alcohol consumption status, number of children, menopausal status, age (year) when menstrual period became regular (< 13, 13–14, 15–16, or ≥ 17), family history of breast cancer, total energy intake

## Discussion

In this large prospective cohort study of more than 34,000 female participants from the Singapore Chinese Health Study with over 17 years of follow-up, we found that adherence to a low-carbohydrate diet, particularly one based on plant-derived sources was associated with a modest reduction in breast cancer risk. Specifically, while the association was statistically significant for the total LCD score, the inverse association for plant-based LCD score was borderline significant and no association was observed for animal-based LCD score.

To our knowledge, the current analysis is the first study examining the association between LCD scores and risk of incident breast cancer; thus, finding a comparable study is a challenge. However, a report of 9,621 women with stage I-II breast cancer from two on-going cohort studies (i.e., the Nurses’ Health Study and the Nurses’ Health Study II in the US) found a significantly lower risk of overall mortality among women with breast cancer who had greater adherence to the total LCD and plant-based LCD scores but not animal-based CLD score [23]. Though this study has different outcome (i.e., breast cancer survivor/mortality) to ours (i.e., breast cancer incidence), their findings appear to be consistent with what we found the current analysis.

Our findings of an inverse association for plant-based LCD and risk of breast cancer may be related to a diet with carbohydrates from sources of vegetables, fruits or legumes, which provide extensive sources of prevention potentials, including antioxidants [35]. In a prior analysis, we showed that a vegetable-fruit-soy dietary pattern was associated with decreased risk of breast cancer among female participants in the Singapore Chinese Health Study [32]. Specifically, among postmenopausal women, individuals who adhered to this dietary pattern had a significantly and dose–response manner risk reduction of breast cancer (HR = 0.70, 95% CI: 0.51–0.95 for quartile 4 compared with quartile 1). A recent meta-analysis of 17 studies, including three prospective cohort studies and 14 case-controls studies of 12,150 breast cancer cases, found that total allium vegetables (odds ratio-OR = 0.70, 95% CI: 0.49–0.91), garlic (OR = 0.77, 95% CI: 0.61–0.93) and onion (OR = 0.75, 95% CI: 0.53–0.98) consumptions were associated with reduced risk of breast cancer [36]. Another meta-analysis of two prospective cohort studies and 11 case–control studies of 18,673 breast cancer cases also found that high intake of cruciferous vegetables was significantly associated with reduced risk of breast cancer (rate ratio-RR = 0.85, 95% CI: 0.77–0.94) [37].

Our study has several limitations. First, dietary data were collected only at baseline, limiting our ability to account for dietary changes over time. This may have introduced non-differential misclassification, and potentially lead to change the direction of overall estimates of association toward the null [38]. In other words, the true estimates might have been stronger than what we reported herein. Second, we were unable to construct glycemic index and glycemic load scores, two key measures of carbohydrate quality, due to the lack of region-specific data. These indices were primarily developed using Western dietary patterns and may not accurately reflect carbohydrate intake in the Chinese populations [39–41]. However, a meta-analysis of 19 studies with 45,790 breast cancer cases reported that glycemic load (RR = 1.04, 95% CI: 1.00–1.07 per 10 units/d) was significantly associated with increased risk of breast cancer [42]. In addition, our findings may not be generalizable to Western populations, given significant differences in dietary composition—specifically, the higher carbohydrate and vegetable intake and lower intake of animal-derived protein and fat among Chinese adults [43]. We also did not have access to medical and/or pathology reports, thus were unable to obtain information regarding the estrogen receptor (ER), and/or progesterone receptor (PR) status for further stratified analysis. Consequently, we were unable to evaluate this association in specific breast cancer subtypes (i.e., ER + /PR + -most common hormone receptor positive (HR +) status, ER + /PR = , ER-/PR + and triple-negative or ER-/PR-/HER2-) and to better understand mechanistically its etiology; which has great implication to treatment plans according to the hormone receptor statuses. Residual confounding is also a concern due to unmeasured and imperfectly measured lifestyle factors, despite extensive multivariable adjustment. Also, our analysis was based solely on self-reported data. The inclusion of biomarkers (e.g., blood lipids, insulin levels, inflammatory markers, etc.) from the available blood samples could have strengthened the exposure assessment and provided insights into potential biological mechanisms linking plant-based LCD to reduced risk of breast cancer. In addition, because the risk estimates for total LCD (*P*_*trend*_ = 0.05) and plant-based LCD (*P*_*trend*_ = 0.06) were of borderline statistical significance, our finding should be interpreted cautiously. Since we did not control for multiple comparison due to the nature of exploratory, our finding should be also interpreted cautiously because some findings in the stratified analysis could be by chance. Finally, due to limitations in registry data, we lacked information on tumor grade and stage, preventing subgroup analyses by cancer aggressiveness.

Despite these limitations, our study has notable strengths. To our knowledge, this is the first prospective study to examine the association between LCD scores and breast cancer risk. The cohort design enabled us to assess dietary and lifestyle exposures before cancer diagnosis, minimizing recall bias and reverse causation. The long follow-up period provided sufficient statistical power to detect associations. In addition, the detailed baseline data allowed us to control for comprehensive and potential confounding factors in the multivariable regression models.

In summary, we found that adherence to a low-carbohydrate diet, particularly dietary pattern based on plant-derived sources, was modestly associated with reduced risk of breast cancer in the Chinese Singaporean population. These results suggest that dietary patterns emphasizing plant-based rather than animal-based sources of protein and fat may play an important role in the primary prevention of breast cancer.

## Supplementary Information

Below is the link to the electronic supplementary material.

Supplementary Material 1

## Abbreviations

BMI: Body mass index
CI: Confidence interval
HR: Hazard ratio
LCD: Low-carbohydrate diet
NHS: Nurses’ Health Study
SCHS: Singapore Chinese Health Study
SD: Standard deviation
