# Supplementary material for: Low-carbohydrate diet score and risk of breast cancer: findings from a prospective cohort study
Source: Breast Cancer. 2026 Feb 4;33(2):448–58. doi: 10.1007/s12282-026-01826-7 (PMC12960405; doi:10.1007/s12282-026-01826-7)
Supplement: Supplementary file 1 — Supplementary Material 1 [file 12282_2026_1826_MOESM1_ESM.docx]

**Supplementary Table 1. Baseline characteristics and daily intake of nutrient by participants in the highest compared with the lowest quartiles of total, animal-based, and plant-based low-carbohydrate diet scores (LCDs)**

**The Singapore Chinese Health Study, 1993-2015**

|  | **Total LCD** | | | **Animal-based LCD** | | | **Plant-based LCD** | | |
| --- | --- | --- | --- | --- | --- | --- | --- | --- | --- |
|  | **Quartile 1**  **N, %** | **Quartile 4**  **N, %** | ***P-value*** | **Quartile 1**  **N, %** | **Quartile 4**  **N, %** | ***P-value*** | **Quartile 1**  **N, %** | **Quartile 4**  **N, %** | ***P-value*** |
| Age, (Mean±SD) | 57.9 ± 8.1 | 55.0 ± 7.7 | <0.001 | 57.8 ± 8.1 | 55.2 ± 7.8 | <0.001 | 58.0 ± 8.1 | 55.0 ± 7.7 | <0.001 |
| Sex |  |  |  |  |  |  |  |  |  |
| Female | 7,377 (50.0) | 9,056 (61.2) | <0.001 | 8,872 (55.4) | 8,983 (55.0) | 0.15 | 7,752 (49.2) | 10,243 (62.6) | <0.001 |
| Male | 7,382 (50.0) | 5,731 (38.8) |  | 7,150 (44.6) | 7,351 (45.0) |  | 8,008 (50.8) | 6,116 (37.4) |  |
| Highest Level of Education |  |  |  |  |  |  |  |  |  |
| No formal education | 4,735 (32.1) | 3,446 (23.3) | <0.001 | 5,238 (32.7) | 3,708 (22.7) | <0.001 | 5,248 (33.3) | 3,679 (22.5) | <0.001 |
| Primary school | 6,766 (45.8) | 6,228 (42.1) |  | 7,129 (44.5) | 7,119 (43.6) |  | 7,273 (46.2) | 6,784 (41.5) |  |
| Secondary school or higher | 3,258 (22.1) | 5,113 (34.6) |  | 3,655 (22.8) | 5,507 (33.7) |  | 3,239 (20.5) | 5,896 (36.0) |  |
| Dialect |  |  |  |  |  |  |  |  |  |
| Cantonese | 6,910 (46.8) | 6,890 (46.6) | 0.07 | 7,345 (45.8) | 7,623 (46.7) | 0.49 | 7,024 (44.6) | 7,854 (48.0) | <0.001 |
| Hokkien | 7,849 (53.2) | 7,897 (53.4) |  | 8,677 (54.2) | 8,711 (53.3) |  | 8,736 (55.4) | 8,505 (52.0) |  |
| Weekly Physical Activity |  |  |  |  |  |  |  |  |  |
| No | 10,013 (67.8) | 9,983 (67.5) | 0.006 | 10,903 (68.0) | 11,056 (67.7) | <0.001 | 10,841 (68.8) | 10,748 (65.7) | <0.001 |
| Yes | 4,746 (32.2) | 4,804 (32.5) |  | 5,119 (32.0) | 5,278 (32.3) |  | 4,919 (31.2) | 5,611 (34.3) |  |
| Smoking Status |  |  |  |  |  |  |  |  |  |
| Never Smoker | 9,694 (65.7) | 10,829 (73.2) | <0.001 | 11,182 (69.8) | 11,166 (68.4) | 0.004 | 10,020 (63.6) | 12,319 (75.3) | <0.001 |
| Ever Smoker | 5,065 (34.3) | 3,958 (26.8) |  | 4,840 (30.2) | 5,168 (31.6) |  | 5,740 (36.4) | 4,040 (24.7) |  |
| Alcohol Consumption |  |  |  |  |  |  |  |  |  |
| Non-Drinker/Monthly drinker | 13,259 (89.8) | 13,212 (89.9) | <0.001 | 14,717 (91.8) | 14,057 (86.1) | <0.001 | 14,113 (89.5) | 14,454 (89.4) | <0.001 |
| Weekly drinker | 1,096 (7.4) | 1,225 (8.3) |  | 984 (6.1) | 1,626 (9.9) |  | 1,194 (7.6) | 1,385 (8.5) |  |
| Daily drinker | 404 (2.7) | 350 (2.4) |  | 321 (2.0) | 651 (4.0) |  | 453 (2.9) | 520 (3.2) |  |
| Coffee drinking status |  |  |  |  |  |  |  |  |  |
| Non-drinker/monthly/weekly | 4,224 (28.8) | 4,711 (31.9) | <0.001 | 4,713 (39.4) | 5,034 (30.8) | 0.01 | 4,537 (28.8) | 5243 (32.0) | <0.001 |
| 1 cup/day | 5,121 (34.7) | 5,565 (37.6) |  | 5,824 (36.3) | 5,801 (35.5) |  | 5,300 (33.6) | 6199 (37.9) |  |
| 2-3 cups/day | 4,780 (33.0) | 4,013 (27.1) |  | 4,874 (30.4) | 4,836 (29.6) |  | 5,189 (32.9) | 4418 (27.0) |  |
| ≥4 cups/day | 634 (4.3) | 498 (3.4) |  | 611 (3.8) | 663 (4.1) |  | 734 (4.7) | 499 (3.0) |  |
| History of Diabetes |  |  |  |  |  |  |  |  |  |
| No | 13,717 (92.9) | 13,111 (88.7) | <0.001 | 14,773 (92.2) | 14,644 (89.7) | <0.001 | 14,587 (92.6) | 14,667 (89.7) | <0.001 |
| Yes | 1,042 (7.1) | 1,676 (11.3) |  | 1,249 (7.8) | 1,690 (10.3) |  | 1,173 (7.4) | 1,692 (10.3) |  |
| Family History of breast cancer |  |  |  |  |  |  |  |  |  |
| No | 8327 (99.0) | 9830 (98.6) | 0.09 | 8778 (98.9) | 8859 (98.6) | 0.11 | 8887 (99.1) | 8636 (98.6) | 0.0003 |
| Yes | 86 (1.0) | 106 (1.4) |  | 93 (1.1) | 121 (1.4) |  | 79 (0.9) | 126 (1.4) |  |
| Use of hormone replacement therapy |  |  |  |  |  |  |  |  |  |
| No | 8059 (95.8) | 8415 (92.9) | <0.001 | 8487 (95.7) | 8373 (93.2) | <0.001 | 8591 (95.8) | 8152 (93.0) | <0.001 |
| Yes | 354 (4.2) | 639 (7.1) |  | 384 (4.3) | 687 (6.8) |  | 375 (4.2) | 610 (7.0) |  |
| Age at menarche (Mean±SD) | 14.6±1.8 | 14.2±1.8 | 0.04 | 14.6±1.8 | 14.2±1.8 | 0.02 | 14.7±1.8 | 14.2±1.8 | 0.43 |
| Age when period became regular (Mean±SD) | 14.3±3.0 | 13.9±3.2 | 0.18 | 14.3±3.1 | 14.9±3.2 | 0.11 | 14.4±3.1 | 13.9±3.2 | 0.51 |
| Number of children (Mean±SD) |  |  |  |  |  |  |  |  |  |
| 0 | 641 (7.6) | 605 (6.7) | <0.001 | 667 (7.5) | 651 (7.2) | <0.001 | 641 (7.1) | 618 (7.0) | <0.001 |
| 1-2 | 2095 (24.9) | 2802 (30.9) |  | 2263 (25.5) | 2769 (30.8) |  | 2163 (24.1) | 2821 (32.2) |  |
| 3-4 | 2947 (35.0) | 3493 (38.6) |  | 3135 (35.3) | 3405 (37.9) |  | 3141 (35.0) | 3408 (38.9) |  |
| ≥5 | 2730 (32.4) | 2154 (23.8) |  | 2806 (31.6) | 2155 (24.0) |  | 3021 (33.7) | 1915 (21.9) |  |
| Age at first child (Mean±SD) | 24.5±4.7 | 24.7±4.6 | 0.11 | 24.5±4.7 | 24.7±4.7 | 0.58 | 24.4±4.7 | 24.9±4.6 | 0.16 |
| Menopausal status |  |  |  |  |  |  |  |  |  |
| Still menstruating | 1743 (20.7) | 3156 (34.9) | <0.001 | 1911 (21.5) | 3057 (34.0) | <0.001 | 1840 (20.5) | 3120 (35.6) | <0.001 |
| Natural | 5951 (78.7) | 5005 (55.3) |  | 6198 (69.9) | 5077 (56.5) |  | 6363 (71.0) | 4793 (54.7) |  |
| Other | 719 (8.6) | 893 (9.9) |  | 762 (8.6) | 846 (9.4) |  | 763 (8.5) | 849 (9.7) |  |
| Age at menopausal (Mean±SD) | 49.5±4.3 | 49.1±4.3 | 0.43 | 49.7±4.3 | 49.2±4.3 | 0.83 | 49.5±4.3 | 49.1±4.4 | 0.26 |
| BMI, kg/m (Mean±SD) | 23.0±3.2 | 23.3±3.4 | <0.001 | 23.1±3.2 | 23.2±3.3 | 0.009 | 23.0±3.2 | 23.3±3.3 | <0.001 |
| Total Energy Intake (kcal/day) (Mean±SD) | 1,466.5±501.8 | 1,685.4±632.1 | <0.001 | 1,433.7±485.5 | 1,705.0±640.0 | <0.001 | 1,464.4±508.8 | 1,668.9±619.0 | <0.001 |
| Carbohydrate (gr/day) (Mean±SD) | 250.2±84.3 | 209.9±77.0 | <0.001 | 241.2±82.2 | 216.9±79.9 | <0.001 | 242.4±84.0 | 216.6±78.2 | <0.001 |
| Dietary fiber (gr/day) (Mean±SD) | 11.4±5.4 | 13.8±6.2 | <0.001 | 12.2±5.8 | 13.0±5.9 | <0.001 | 10.7±5.0 | 14.8±6.4 | <0.001 |
| Total fat (gr/day) (Mean±SD) | 30.0±11.8 | 59.9±25.0 | <0.001 | 31.1±12.8 | 58.3±25.0 | <0.001 | 31.7±13.3 | 57.1±24.2 | <0.001 |
| Animal fat (gr/day) (Mean±SD) | 9.3±5.5 | 21.8±12.0 | <0.001 | 8.0±4.3 | 23.6±11.5 | <0.001 | 11.8±7.3 | 18.0±10.9 | <0.001 |
| Plant fat (gr/day) (Mean±SD) | 20.6±8.3 | 38.1±16.0 | <0.001 | 23.1±10.4 | 34.7±15.7 | <0.001 | 19.9±7.8 | 39.1±15.8 | <0.001 |
| Saturated fat (gr/day) (Mean±SD) | 10.5±4.6 | 21.2±9.6 | <0.001 | 10.6±4.8 | 21.1±9.6 | <0.001 | 11.9±5.6 | 19.2±9.4 | <0.001 |
| Monunsaturated fat (gr/day) (Mean±SD) | 10.0±4.1 | 20.4±8.8 | <0.001 | 10.3±4.3 | 20.1±8.8 | <0.001 | 10.9±4.8 | 19.0±8.5 | <0.001 |
| Polyunsataturated fat (gr/day) (Mean±SD) | 6.2±3.0 | 11.9±6.0 | <0.001 | 6.8±3.6 | 11.2±5.8 | <0.001 | 5.6±2.4 | 12.6±5.9 | <0.001 |
| Total Protein (gr/day) (Mean±SD) | 45.7±16.5 | 75.5±28.1 | <0.001 | 45.2±16.1 | 75.0±28.0 | <0.001 | 48.5±17.8 | 71.1±27.5 | <0.001 |
| Animal protein (gr/day) (Mean±SD) | 19.8±9.3 | 45.9±18.9 | <0.001 | 18.5±8.2 | 47.0±18.1 | <0.001 | 24.3±11.6 | 39.2±18.8 | <0.001 |
| Plant protein (gr/day) (Mean±SD) | 7.3±4.1 | 15.8±8.2 | <0.001 | 8.7±5.6 | 13.7±7.5 | <0.001 | 6.2±3.0 | 17.6±8.0 | <0.001 |

Means and standard deviation are calculated for continuous variables. Weekly physical activity including strenuous physical activity and/or vigorous work

Abbreviations: BMI: body mass index; LCD: low-carbohydrate diet; SD: standard deviation.
